# General practitioners’ perspectives on barriers and facilitators of the implementation of the ANTIBIORESIST antibiotic stewardship audit and feedback intervention: a qualitative interview study among high prescribers

**DOI:** 10.1093/jacamr/dlag197

**Published:** 2026-09-08

**Authors:** Mehdi El Azrak, Aurélie Bocquier, Cécile Féry, Céline Pulcini, Maïa Simon, Laetitia Ricci, Nelly Agrinier

**Affiliations:** CHRU de Nancy, Inserm, Université de Lorraine, CIC, Epidémiologie Clinique, F-54000 Nancy, France; Université de Lorraine, Inserm, INSPIIRE, F-54000 Nancy, France; CHRU de Nancy, Inserm, Université de Lorraine, CIC, Epidémiologie Clinique, F-54000 Nancy, France; Université de Lorraine, Inserm, INSPIIRE, F-54000 Nancy, France; Université de Lorraine, CHRU-Nancy, Centre Régional en Antibiothérapie du Grand Est AntibioEst, F-54000 Nancy, France; Université de Lorraine, Inserm, INSPIIRE, F-54000 Nancy, France; Université de Lorraine, CHRU-Nancy, Département Méthodologie Promotion Investigation, F-54000 Nancy, France; CHRU de Nancy, Inserm, Université de Lorraine, CIC, Epidémiologie Clinique, F-54000 Nancy, France; Université de Lorraine, Inserm, INSPIIRE, F-54000 Nancy, France; CHRU de Nancy, Inserm, Université de Lorraine, CIC, Epidémiologie Clinique, F-54000 Nancy, France; Université de Lorraine, Inserm, INSPIIRE, F-54000 Nancy, France

## Abstract

**Background:**

Audit and feedback (A&F) interventions are critical for improving antibiotic prescribing practices among general practitioners (GPs) but more knowledge about the optimal ways to deliver them is needed. The French ANTIBIORESIST programme delivers individualized A&F based on proxy indicators estimating the appropriateness of antibiotic prescriptions.

**Objectives:**

This study aimed at exploring high prescribing GPs’ perspectives on barriers to and facilitators of implementation (i.e. acceptability, demand and integration into daily practice) of the ANTIBIORESIST A&F intervention.

**Methods:**

Semi-structured interviews were conducted (May–October 2024) with 10 GPs from northeastern France whose prescribing patterns were the least compliant with guidelines and who had received ANTIBIORESIST A&F intervention. The data were analysed thematically based on the Bowen’s feasibility framework.

**Results:**

Twenty-two elements were identified across A&F design, cognitive accessibility, reaction to performance score, perceived legitimacy of the provider and delivery modality. Facilitators included concise, colour-coded feedback, relevance of proxy indicators and in-person delivery. Barriers included misunderstanding and misinterpretation of proxy indicators, the perception that they are not applicable to their own context of practice and scepticism towards the feedback provider. Heterogeneity in interpretation of proxy indicators often hinders acceptability. Integration would be enhanced if feedback were delivered via digital workspaces, yet GPs expressed concerns about coercion and A&F futility.

**Conclusions:**

Addressing cognitive and contextual barriers is essential for optimizing A&F interventions. Enhancing the clarity, legitimacy of the feedback provider and delivery methods, coupled with supportive co-interventions, may strengthen the impact on antibiotic prescribing in primary care.

## Background

Antibiotic resistance constitutes a critical threat to public health, as demonstrated by a marked increase in mortality associated with multidrug-resistant bacteria worldwide over the past three decades.^1^ In France alone, antibiotic-resistant infections accounted for 103 672 cases and 4480 deaths in 2019.^2^ The emergence and spread of antibiotic-resistant bacteria are primarily driven by the misuse and overuse of antibiotics.^3,4^ Notably, general practitioners (GPs) significantly contribute to antibiotic consumption and were responsible for 74.6% of all outpatient antibiotic prescriptions in France in 2023.^5^

Audit and feedback (A&F) are quality improvement strategies that involve measuring professional performance, with the results subsequently being provided to clinicians and/or their teams to encourage positive change related to the implementation of evidence-based practices in health service settings.^6–8^ To promote antibiotic stewardship (ABS) within outpatient primary care, numerous studies, including a recent meta-analysis, have demonstrated that providing GPs with individualized feedback on their antibiotic prescribing patterns can substantially reduce antibiotic prescribing rates.^6,9–11^ However, some investigations report little or no effect of these A&F interventions,^12,13^ and heterogeneity was substantial in the meta-analysis. A&F should be regarded as a complex intervention whose effectiveness is influenced by multiple interacting components and the specific context of implementation; thus, its evaluation requires adherence to established methodological frameworks as proposed by the Medical Research Council.^14,15^ In addition, according to the US CDC, which outlines core elements for outpatient ABS programmes, the impact of A&F on optimizing prescribing practices is likely enhanced when A&F is combined with co-interventions such as educational resources, peer support and monitoring.^16^

The ANTIBIORESIST ABS programme was developed to optimize antibiotic prescribing among French GPs. This programme comprises individualized A&F on antibiotic prescribing practices (please see the details in the Methods section) and three co-interventions (an online repository of training modules, an ABS toolbox for infection management and academic detailing).^17,18^ The ANTIBIORESIST-EVAL research project aimed to assess the effectiveness and implementation of this programme and first included a pilot randomized controlled trial focusing on the A&F intervention. Using a mixed-method approach, this pilot study will help refine the A&F intervention and will be succeeded by a larger-scale quasi-experimental study.

As part of the qualitative investigations of the ANTIBIORESIST-EVAL pilot study, we sought to examine high prescribing GPs’ perspectives on barriers to and facilitators of implementation (i.e. acceptability, demand and integration into daily practice) of the A&F intervention.

## Methods

### Intervention

**Figure 1.**
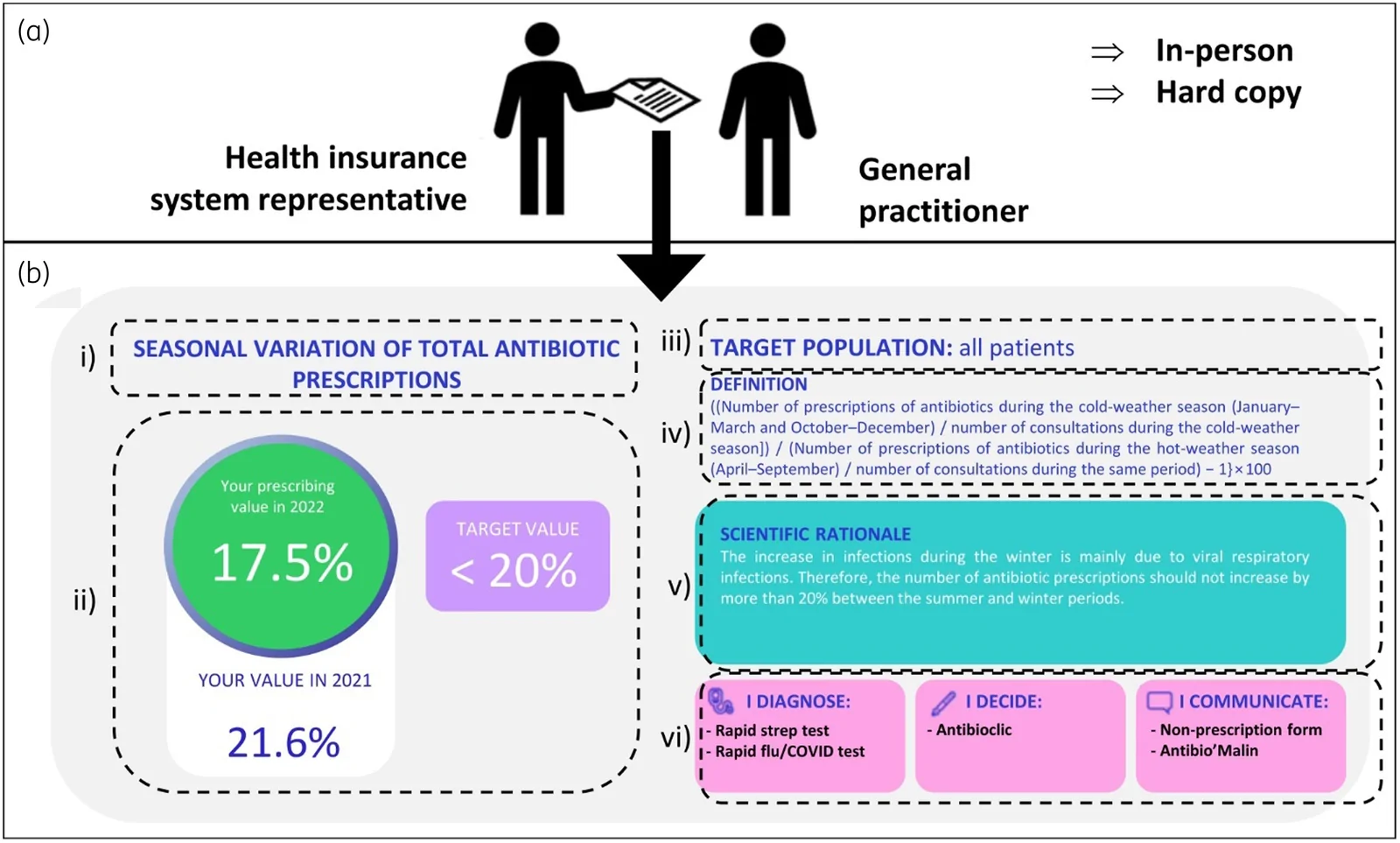
Illustration of the individualized ANTIBIORESIST A&F intervention and features. (a) Representation showing the feedback intervention of the programme: a French health insurance system representative gives a hard copy of the individualized feedback to the GP’s practice in-person. (b) Extract of the fictitious feedback translated into English with an example of a proxy indicator including (i) the title of the proxy indicator; (ii) the score achieved by the GP for the proxy indicator, with the last year’s value displayed below and the target value on the right; (iii) the target population; (iv) the calculation method definition; (v) the scientific rationale; and (vi) the tools available for the GP to help her or him improve her or his score in the future. A&F, audit and feedback; GP, general practitioner.

The A&F intervention of the ANTIBIORESIST ABS programme (see Template for Intervention Description and Replication, TIDieR, Checklist in Additional File 1, available as Supplementary data at *JAC-AMR* Online) is based on the Clinical Performance Feedback Intervention Theory (CP-FIT) for designing, implementing and evaluating feedback in health care.^19^ This intervention has been developed since 2020, informed by the results of a diagnostic phase including (i) a survey among GPs; (ii) semi-structured interviews with delegates and physicians from the health insurance system and GPs; (iii) a literature review of guidelines to produce effective feedback interventions; and (iv) an inventory of feedback tools used in French primary care.^20^ The A&F intervention of the ANTIBIORESIST ABS programme was then refined with a scientific committee (including GPs, ID and public health specialists and regional ABS experts and health insurance representatives), with the aim of scaling it up at the regional and then national levels if it proves successful. Its content is based on 11 proxy indicators that were initially developed by Thilly *et al.*^21^ and further validated by Simon *et al*.^22^ These proxy indicators are designed to estimate the appropriateness of GPs’ antibiotic prescriptions according to national guidelines using aggregated reimbursement data from the French health insurance system. They are calculated at the level of the individual GP, on the basis of their prescribing patterns, and are then compared against predefined target values (see Figure 1). These target values were established in accordance with national antibiotic prescribing guidelines, considering seasonal fluctuations in the incidence of infectious diseases and variability among patients (list of proxy indicators available in Additional File 2). An aggregated score reflecting the proportion of proxy indicators falling within the target values was also calculated. Individualized feedback was provided to the GPs in hard-copy format during an on-site visit conducted by a representative of the French health insurance system during the first semester of 2024 (see Figure 1). Each GP received it only once, as its optimal frequency will be defined based on our research results, while considering the available resources of the health insurance system. In France, the health insurance system routinely provides GPs with feedback on the volume of their antibiotic prescriptions.

### Study design and settings

The ANTIBIORESIST-EVAL pilot study included GPs from the Grand Est region (northeastern France) who were the least compliant with national guidelines related to antibiotic prescriptions (i.e. those who had the greatest potential for improvement). These GPs were identified using a clustering method developed by Simon *et al*.^23^ which is based on the aggregated score calculated from the 11 proxy indicators for the year 2022 for all the GPs from the Grand Est region (*n* = 4300). In total, 1843 (43%) participants were included and were randomly allocated in a 4:1 ratio to either receive an A&F intervention (intervention group) or to serve as controls (control group) during the first semester of 2024. The results of this randomized controlled trial will be published in a separate article.

Within the ANTIBIORESIST-EVAL pilot study, as part of the qualitative approach, we conducted semi-structured interviews with GPs randomized into the intervention group. We reported the study in alignment with the Consolidated Criteria for Reporting Qualitative Research (COREQ) guidelines (Additional File 3).^24^

### GP sampling

All the GPs in the intervention group and who had received feedback (*n* = 987) were sent email invitations by the health insurance system to participate in interviews.

Given the challenges in providing clear evidence to support claims of data saturation, the concept of data sufficiency based on information power has emerged. According to this concept, when the research objective is narrowly defined, the sample can be selected for a specific purpose, making large sample sizes unnecessary; under such conditions, a sample of 10 or fewer respondents may be sufficient.^25^ Given that the interviewed GPs had substantial familiarity with the topic, our study fulfilled these criteria. This approach particularly depends on the researchers’ aims; for example, studies aiming to describe a phenomenon may require a smaller number of interviews than those seeking to develop theory.^25,26^ Based on these considerations, a sample size of 10 participants was deemed adequate to meet the study objective. Young *et al*. provided reassuring evidence that reliable results can be obtained with small samples, as themes and codes can be obtained from ten semi-structured interviews.^27^

### Data collection

The interviews were conducted by following an interview guide (Table 1). L.R. (PhD, health psychologist) and C.F. (MA, sociologist) developed the initial draft. Afterward, C.P. (MD, PhD, infectious disease specialist), N.A. (MD, PhD, public health specialist) and A.B. (PhD, public health specialist) refined the guide. The guide was not pilot tested due to time constraints. Data collection was performed between 29 May 2024 and 2 October 2024 by C.F. (MA, female sociologist), who is trained in qualitative methods and who had no prior assumptions about GP practices and no prior relationship with them. The interviews were conducted via videoconference or by telephone. No one other than the sociologist or the participant was present during the interviews.

**Table 1.** Interview guide themes related to the audit and feedback intervention with example questions

Theme	Example of questions
Feedback delivery modalities	How should an ideal feedback delivery visit be conducted? In what ways could the visit be improved? Looking ahead, how would you prefer to receive your feedback (e.g. email, postal mail)? Which delivery method would best suit your needs?
Feedback content relevance	What is your opinion of the aggregated score presented in your feedback? How do you perceive the help or advice associated with each proxy indicator? What do you think of the visual design of the feedback? How relevant do you find the eleven proxy indicators included in your feedback? What does [each proxy indicator] mean to you?
Feedback delivery frequency	How frequently would you prefer to receive feedback?
Feedback impact	Did the feedback influence your intention to modify your antibiotic prescribing practices?
Areas for improvement	What could be improved in the feedback?

For each GP interviewed, data were collected on their sex, age, years of clinical experience and the geographic location of their practice, including department and city.

During the interviews, we asked the GPs to respond to an example of feedback by reviewing it aloud in real time with the interviewer. We used fictitious feedback based on a standard template using data from a fictitious GP to guide the discussion. We used such a method because French regulations prevented the health insurance system from sharing participants’ prescription data with the research team and because it was uncertain whether GPs would have their own feedback available during interviews. Moreover, this fictitious feedback ensured a single dataset, enabling more consistent comparisons of GPs’ responses.

No interviews were repeated, and no field notes were taken during or after the interviews. All the interviews were audio-recorded and transcribed anonymously.

### Data analysis

The CP-FIT framework was used in this study as the overarching theoretical framework to develop the A&F intervention and provides a conceptual basis to explore the underlying causal mechanisms by which the intervention may influence intention to change practices and then actual practices. However, at this early stage of intervention development and before potential refinement, we did not aim at exploring these mechanisms in depth but rather addressing the question: ‘Can the A&F work?’. Therefore, we used the Bowen’s feasibility framework to guide the analysis as it is particularly well-suited for addressing this type of research question across a wide range of health interventions.^28^ Following the review of the initial three interviews, a coding tree was developed focusing on three areas of focus from Bowen’s framework: acceptability, demand and integration into daily practice (Table 2). We considered the other areas of focus within Bowen’s framework (e.g. adaptation, practicality) of limited relevance for our pilot study, as they relate more to mature or already established interventions within specific contexts, and our data were not suited to exploring them. The thematic analysis was inductive–deductive as the main themes were derived from Bowen’s framework, while subthemes were developed inductively from the data.

**Table 2.** Definition of areas of focus on A&F implementation used in the coding tree

Area of focus	Definition
Acceptability to recipients	To what extent was the A&F intervention perceived as appropriate, satisfactory or appealing by the participants?
Demand (perceived usefulness)	To what extent was the A&F intervention likely to be utilized in practice?
Integration into daily practice	To what extent could the A&F be incorporated into existing clinical and organizational systems?

Areas of focus based on Bowen’s feasibility framework.

A&F, audit and feedback.

The interviews were independently double-coded by a health sociologist (C.F.) and a male public health resident (M.E.A.). Any discrepancies in coding were resolved through iterative discussion until a Cohen’s kappa coefficient exceeding 0.8 was achieved, ensuring strong inter-rater reliability. Data analysis was performed using NVivo version 14 software.

### Ethics approval and consent to participate

This research was conducted in accordance with the MR-004 methodological guidelines dated 3 May 2018, which were approved by the French Data Protection Authority (Commission Nationale de l'Informatique et des Libertés, CNIL). The University of Lorraine has complied with these guidelines through a declaration made by the project coordinator to its CNIL-registered data protection officer on 19 December 2023, under registration number 2023/301. Ethical approval for the present study was obtained from the Inserm Institutional Review Board (*Comité d’évaluation éthique de l’Inserm*—CEEI/IRB00003888). It was conducted in compliance with the applicable French regulatory framework, the Declaration of Helsinki and Good Clinical Practice guidelines. The participants received an information note and could ask for details before the beginning of the interview. We also collected oral consent before the start of the interviews. Written consent was not necessary because the data we collected referred to the humanities and was not linked to health data of professionals. In this context, French regulations encourage oral consent after clear written information has been provided. Participating GPs were offered financial remuneration amounting to €50 as compensation for foregone consultation fees during the interview period.

## Results

Ten GPs who volunteered for this qualitative approach of the ANTIBIORESIST-EVAL pilot study were subsequently included. The interviews lasted an average of 36 min. Eight respondents were male, and two were female, with a mean age of 52.2 years and an average clinical experience of 23.5 years (Table 3). The sample was distributed across rural (*n* = 5), semi-urban (*n* = 3) and urban (*n* = 2) practice settings, encompassing 7 of the 10 departments within the Grand Est region. Consequently, the sampling met the standard criteria for maximum variation in the collected data (see detailed characteristics of the sample in Additional File 4).

**Table 3.** Characteristics of the included GPs

Characteristic	*n* = 10^a^
Sex	
Female	2
Male	8
Age (years)	52.2 (10.9)
Professional experience (years)	23.5 (10.3)
Location	
Rural	5
Semi-urban	3
Urban	2

GP, general practitioner.

^a^ *n*; mean (SD).

**Figure 2.**
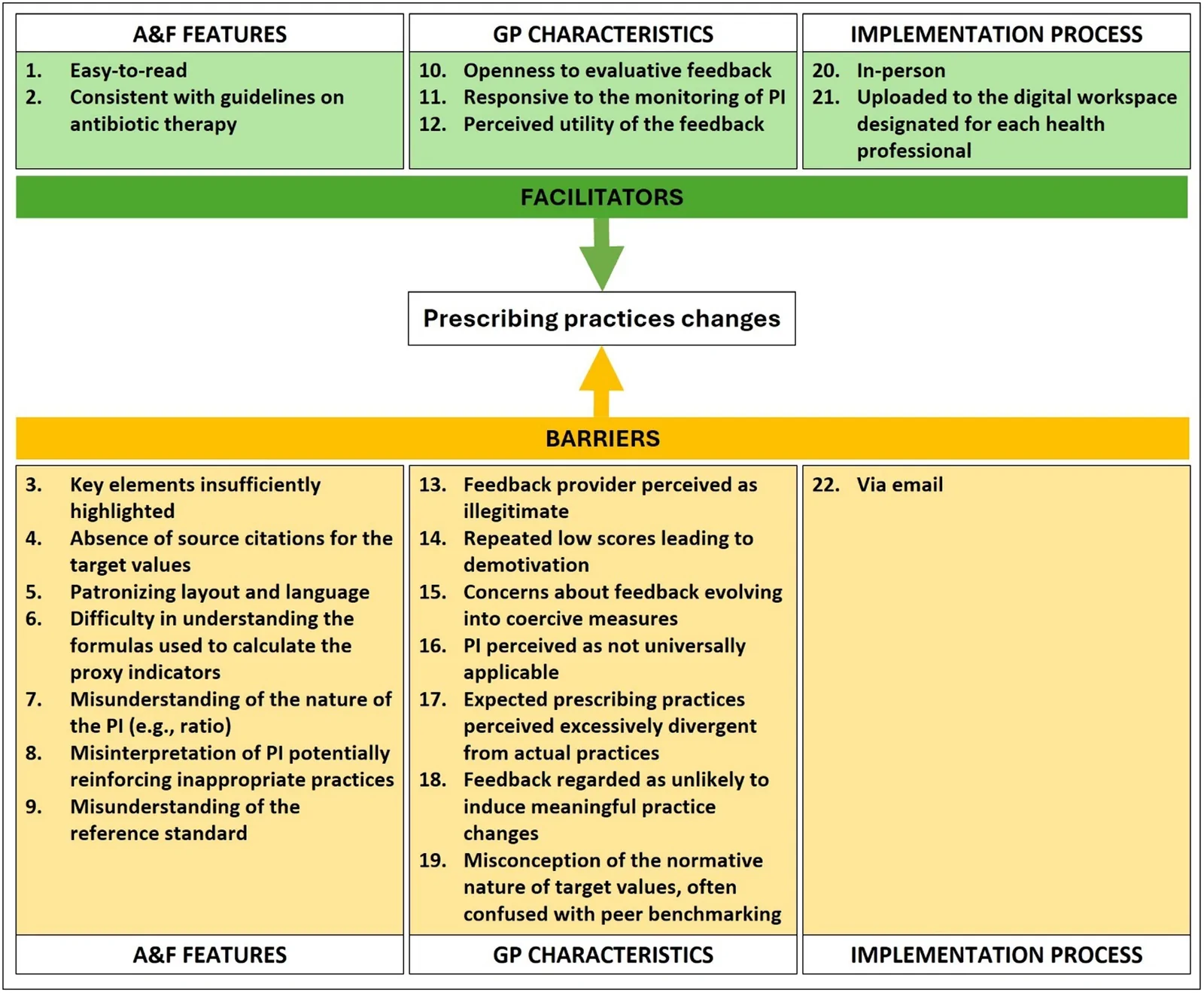
Logic model of factors^a^ influencing changes in GPs’ prescribing practices during the ANTIBIORESIST A&F intervention. ^a^Numbers in parentheses refer to the factor numbers as listed in Table 4. A&F, audit and feedback; GP, general practitioner; PI, proxy indicator.

Table 4 presents 22 elements identified as having potentially influenced the impact of the A&F on the GPs’ intention to change their antibiotic prescribing practices clustered into the themes of acceptability, demand and integration of the A&F. Furthermore, Figure 2 illustrates the logic model of these 22 elements as grouped according to the (i) A&F features, (ii) GP characteristics and (iii) implementation process.

**Table 4.** A&F implementation analysis grid, with the 22 identified elements classified as facilitators or barriers and clustered into their higher-level themes and subthemes

Theme	Subtheme	Facilitator if…	Barrier if…
Acceptability	Design and linguistic features	(1) …easy-to-read	(2) …patronizing layout and language (3) …key elements insufficiently highlighted
	Perceived relevance of proxy indicators relevance	(4) …consistent with guidelines on antibiotic therapy	(5) …expected prescribing practices perceived as excessively divergent from actual practices (6) …proxy indicators perceived as not universally applicable
	Cognitive accessibility of proxy indicators	—	(7) …difficulty in understanding the formulas used to calculate the proxy indicators (8) …misunderstanding of the nature of the proxy indicator (proportion, ratio, etc.) (9) …misinterpretation of proxy indicator potentially reinforcing inappropriate practices
	Cognitive accessibility of target values for proxy indicators	—	(10) …misconception of the normative nature of target values, often confused with peer benchmarking (11) …misunderstanding of the reference standard (12) …absence of source citations for the target values
	Reaction performance scores	(13) …openness to evaluative feedback	(14) …repeated low scores leading to demotivation
	Perceived legitimacy and trustworthiness of the feedback provider	—	(15) …concerns about feedback evolving into coercive measures (16) …feedback provider perceived as illegitimate
Demand (perceived usefulness)		(17) …perceived utility of feedback *(*18) …responsive to the monitoring of proxy indicator	(19) …feedback regarded as unlikely to induce meaningful practice changes
Integration	Feedback delivery modalities	(20) …in-person (21) …uploaded to the digital workspace designated for each health professional	(22) …via email

A&F, audit and feedback.

### Key identified elements shaping feedback acceptability

#### Design and linguistic features

The principal determinant of acceptability was the readability of the feedback, which was improved by increased conciseness and the use of colour coding (Table 5, Quotes 1 and 2).

**Table 5.** Selection of the most illustrative verbatims from general practitioners’ interviews

Themes/Subthemes	Quotes	Verbatim
Acceptability Design and linguistic features	1	It’s very clear and concise. Because to be honest, we get a lot of documents like that, and we often skim read them. So, it’s good that there are lots of colors like this with fairly simple inserts, it’s nice (GP5).
	2	Look, the color codes speak for themselves. Red/green, you know immediately what they mean. No, I find that afterwards, the explanations, ‘populations concerned, scientific rationale’, the color codes make it easy to distinguish each paragraph, and then the short, concise recommendations, you know exactly what to do (GP1).
	3	And there’s red, sometimes? […] it can be a bit off-putting for some (GP8).
	4	I read ‘Better prescribing today means keeping it simple. ‘Well, that’s aimed at doctors. […] It’s a bit childish like that, maybe. So, some people will say ‘yes, but it’s okay, I’m not stupid (GP4).
	5	[About the display of the target population for a proxy indicator] It’s very small, it should actually be in the title (GP4).
Acceptability Perception of proxy indicators relevance	6	[About the proxy indicators] They are quite logical. No, there’s no problem; I agree insofar as, anyway, it’s more or less… it’s the usual antibiotic therapy guidelines’ (GP3).
	7	These numbers have to be interpreted according to epidemiology (…) This winter we had a collective waterborne disease, with a lot of Campylobacter, and we were forced to prescribe antibiotics (GP7).
	8	We took them one by one [talking about the indicators] and I said, ‘Here I can do it, here I can’t do it,’., or ‘Here I’m pretty much on the right track,’ or ‘Here I don’t agree,’ because there are times when just because everyone jumps in the river doesn’t mean I’m going to jump in the river (GP3).
Acceptability Cognitive accessibility of proxy indicators	9	The number of prescriptions for antibiotics in the summer, over the number of consultations over the same period, minus one. Why ‘minus one’? […] It’s complicated. […] It seems too complicated, this thing (GP10).
	10	Does the target mean that more than 5% of people will be prescribed an antibiotic or not? No, what does it mean? What are the percentages? Because frankly, if you have a bladder infection or a urinary tract infection, it’s 100% antibiotics’ (GP9).
	11	[About the proxy indicator for seasonal variation in antibiotic prescriptions] This is perhaps more relevant because it takes into account the seasonality of infections. We will clearly have more prescriptions in winter than in summer, so that's not bad (GP7).
Acceptability Cognitive accessibility of target values	12	Is the target relative to a group of doctors? (GP9).
	13	That surprised me a lot, because when it comes down to it, that’s how the social security always works: it compares everyone’s prescriptions (GP1).
	14	Telling us ‘The target is less than 10%’ means in our minds that we have to use less than 10% of antibiotics. I find that a bit misleading in the sense that we can be at 11% as well as 9%. If you like, if someone needs an antibiotic, we’re not going to base it on the target (GP9).
	15	So how were these indicators taken? How were they taken? With what? With which guidelines? And I'll tell you something else: the guidelines of the French urology society, for example, are not the same as those of the European urology society, which are different again from those of the American societies. (…) If you ask me, the indicators…let’s have some references!’ (GP2).
Acceptability Reactions to performance scores: engagement versus discouragement	16	Personally, I don’t mind being sent the feedback and being told ‘You're bad in one score’, if I can improve it, why not. I don’t mind that at all. I know that our profession is constantly evolving, we’ve had medical revolutions, we have to keep up with them. So it is by questioning ourselves that we manage to improve our practice (GP3).
	17	If you try to make an effort and you always get ‘negative’ feedback, so to speak, that is, you get feedback that is not negative, but you are told, ‘There you go, you are prescribing too much of this, too much of that, too much of that […].’ The risk is okay, but what’s more, let’s say you’re a doctor, you’re about to retire, after a while you're not going to listen to anything anymore (GP9).
Acceptability Perceived legitimacy and trustworthiness of the feedback provider	18	I think it’s good. Then, you know, we just have to see what they do with it. But that’s social security side, I don’t think that’s what we should be penalized for’ (GP4).
	19	It would have to be something that is purely between doctors or between scientists, pharmacists and so on, and not with organizations that can sanction us for nothing or for little. (…) Our professors (…) are better able than we are to say ‘OK, you’re right or you’re wrong’. They are the only ones who can judge us, not the others (GP6).
Key factors shaping demand for feedback	20	It’s very good, it allows you to see the progress. It can motivate the physician to continue to improve his practice or, on the contrary, if the result isn’t as good, to say: ‘Be careful, what happened here? (GP8).
	21	It would be interesting to have my 2023 (GP2).
	22	I’m not sure that it really changes anything, to be perfectly honest’ (GP5).
Key factors shaping feedback integration into daily practice	23	If we get it by post or email, I’m not sure we’ll read it to be honest. We’ll take a quick look, but we won’t necessarily… it will be less striking, less memorable than when we receive someone directly for an interview (GP5).
	24	By email, I have no idea if it will be opened. Frankly, I don’t know (GP7).
	25	They can send it by email and it can be included, why not, in the indicators on the Ameli account. It’s a place where you can find information (GP7).

GP, general practitioner.

However, negative visual cues, such as the red score highlights, acted as a barrier to feedback acceptability and were perceived as stigmatizing by the recipients (Table 5, Quote 3).

The language employed was also critical; some wordings were misinterpreted when perceived as patronizing (Table 5, Quote 4).

Another impediment to acceptability arose when the presentation inadequately highlighted critical elements, such as the target population associated with a proxy indicator (Table 5, Quote 5).

#### Perception of proxy indicators relevance

A&F acceptability was enhanced when GPs perceived the proxy indicators as consistent with established antibiotic prescribing guidelines (Table 5, Quote 6).

However, a barrier to acceptability emerged when the proxy indicators reflected prescribing expectations perceived as excessively divergent from actual clinical practices. This perception appeared to stem from GPs’ belief that the proxy indicators did not adequately account for specific clinical contexts. Consequently, the GPs often attributed discrepancies in their scores to such contextual factors, including localized outbreaks of pathogens (Table 5, Quote 7).

Marked resistance to certain proxy indicators was also evident when the underlying principle of adherence to the guidelines was challenged. Specifically, one interviewed GP articulated a reluctance to conform to the guidelines systematically, particularly those he perceived as flawed. This stance potentially constituted a significant barrier to the acceptability of A&F interventions designed to enhance prescribing practices through evidence-based standards (Table 5, Quote 8).

#### Cognitive accessibility of proxy indicators

The A&F incorporated the computational methodology for each proxy indicator, using mathematical formulas intended to enhance trustworthiness among GPs and facilitate comprehension. However, these formulas proved difficult to interpret, generating confusion particularly regarding the calculation of seasonal variations involving the term ‘-1’, which ultimately impeded acceptability (Table 5, Quote 9).

Proxy indicator values—including the GPs’ estimated values and target values—are presented as ratios (numerical values), proportions (expressed as positive percentages) or variations (expressed as positive or negative percentages). This heterogeneity in data representation led to misunderstandings among some GPs, such as confusion between proportions and ratios, thereby undermining acceptability. For example, one proxy indicator for assessing antibiotic prescriptions for urinary tract infections in women, which employed a target ratio (the number of conventionally recommended antibiotics prescribed relative to the number of fluoroquinolones prescribed, fluoroquinolones only being recommended in rare cases) exceeding five (to one), was subject to misunderstanding (Table 5, Quote 10).

Some proxy indicators were misinterpreted to such an extent that some GPs appeared to endorse the opposite of what was intended. For instance, the proxy indicator measuring the seasonal variation in the total number of antibiotic prescriptions, which had a target value of less than 20% between summer and winter, was interpreted in the completely opposite manner. One GP firmly believed that the winter season naturally entailed a higher incidence of infections requiring antibiotic treatment, which further impeded her understanding of the proxy indicator (Table 5, Quote 11).

#### Cognitive accessibility of target values

Although the proxy indicators and their corresponding target values were developed in alignment with French guidelines to ensure their normative intent, some GPs misunderstood or misinterpreted these targets, perceiving them not as guideline-based standards but rather as comparative benchmarks of peer practice (Table 5, Quotes 12 and 13).

The acceptability of the A&F was also influenced by GPs’ perceptions of it as disruptive to patient care, particularly when the concept of the reference standard was misunderstood. In some cases, the target value was misinterpreted as an overly restrictive threshold for antibiotic prescription—it was perceived as being imposed without consideration for individual clinical judgment or clearly indicated cases (Table 5, Quote 14).

The absence of visible references or sources used to establish the target values also undermines the acceptability of the A&F. This was especially true in cases in which the clinical guidelines issued by learned societies were inconsistent, leaving GPs uncertain about which standard to rely on (Table 5, Quote 15).

#### Reactions to performance scores: engagement versus discouragement

The inclusion of both an aggregated score based on the 11 proxy indicators and a score for each proxy indicator enhanced the acceptability of the A&F when the GPs were receptive to critique and viewed the scores as constructive tools for professional development (Table 5, Quote 16).

Conversely, when scores were persistently low, this scoring system acted as a barrier, with some GPs perceiving it as discouraging and demotivating (Table 5, Quote 17).

#### Perceived legitimacy and trustworthiness of the feedback provider

Some GPs expressed scepticism towards the institution delivering the A&F—specifically, the French health insurance system (referred to as ‘social security’ in the quotation below). Their concerns centred on the potential shift from informative A&F towards more coercive forms, particularly targeting GPs whose practices diverge from established guidelines (Table 5, Quote 18).

The credibility of the A&F was sometimes questioned, as the provider was perceived as illegitimate because it was less qualified to assess prescribing practices than clinical peers, such as physicians or pharmacists specializing in infectious diseases and ABS. This perception further undermined the acceptability of the A&F (Table 5, Quote 19).

### Key identified elements shaping demand for feedback

The perceived utility of the A&F appeared to underpin its demand among GPs. A&F was regarded as a valuable opportunity to enhance prescribing practices and engendered curiosity regarding the longitudinal evolution of proxy indicator scores (Table 5, Quote 20).

Monitoring these scores with regular A&F was considered engaging by the GPs, thereby reflecting a genuine demand for evaluative tools (Table 5, Quote 21).

Conversely, demand was diminished when the A&F was perceived as ineffectual, with efforts within the intervention viewed as unlikely to result in meaningful changes (Table 5, Quote 22).

### Key identified elements shaping feedback integration into daily practice

When queried about the preferred mode of feedback delivery, the GPs expressed a clear preference for in-person dissemination over postal mail, as the presence of the deliverer allowed for a thorough review of the results (Table 5, Quote 23).

In contrast, the feedback conveyed via email would be less effectively integrated, primarily because of the overwhelming volume of messages GPs received from the French health insurance system for other purposes, which diluted their attention (Table 5, Quote 24).

Integration could further be enhanced by uploading a copy of the feedback directly into the GP’s digital workspace, which would be accessible through secure login on to the French health insurance system platform, Ameli.fr (Table 5, Quote 25).

## Discussion

This study identified 22 elements related to acceptability, demand for and integration of the A&F intervention that may critically influence its effectiveness in improving antibiotic prescribing practices among GPs. Notably, the readability and design of the feedback emerged as determinants of acceptability, whereas negative visual cues and patronizing language hindered acceptability. The alignment of proxy indicators with established, clinically relevant guidelines was essential to fostering trust, while perceived discrepancies between proxy indicators or target values and actual practice contexts often generated resistance. Cognitive accessibility also proved vital; complex computational methodologies and inconsistent data representations led to confusion and misinterpretation, thereby undermining acceptability. Lastly, the credibility of the A&F provider and the delivery modalities shaped acceptability, with a marked preference for in-person communication over methods such as email or postal mail.

### Refining the feedback content and layout to improve cognitive accessibility

Recommendations for A&F interventions emphasize the importance of aligning visual displays with summary messages and reducing extraneous cognitive load for recipients.^29,30^ In accordance with these recommendations, our findings prompted us to make several refinements. For instance, as GPs misinterpreted the target values as comparative benchmarks against their peers’ practices, we clarified how these values were established based on recommendations, particularly by replacing confusing mathematical formulas with the meaning of the target values. We also mitigated the cognitive barrier related to uncertainty about the underlying recommendations by explicitly displaying these recommendations and providing easy access to them through clickable links and QR codes. As a final example, due to the limited visibility of the target population associated with each proxy indicator, we emphasized this information by using a larger font and intuitive icons.

### Preference, trust and challenges in feedback intervention implementation

The preference for in-person delivery over email aligns with the literature, which highlights the substantial workload faced by French GPs. This workload constrains their available time, often resulting in feedback sent by email being overlooked amid the overwhelming volume of daily information.^31,32^ These results are consistent with those of the CP-FIT (please see details in the Methods section), which advocates for ‘pushing’ information to recipients proactively rather than relying on them to ‘pull’ it.^19^ However, it was also suggested that feedback should be available as information that GPs can actively retrieve from their digital workspace on a ‘pull’ approach, confirming that, ideally, antibiotic A&F in primary care should be delivered by multiple strategies.^29,30^

Our findings also raise important questions about the initially defined target audience for the feedback, given the considerable variability in attitudes towards ABS among participants. While there appeared to be a demand for repeated A&F, we also observed scepticism towards the health insurance system, particularly regarding its scientific legitimacy. Jami *et al.* have already highlighted the negative attitudes of French GPs towards the health insurance system, particularly with regard to the normative recommendations perceived as being driven by economic considerations.^33^ Health authorities may be perceived as imposing restrictive practices, with only a minority of French GPs expressing strong trust in public health agencies, while mistrust remains prevalent.^34,35^ In the context of a remuneration system increasingly linked to quality metrics (pay-for-performance) rather than fee-for-service payments,^36^ this mistrust towards the health insurance system appears neither novel nor confined to our study. Given the limited human and economic resources, a pragmatic approach to the next phase of the ANTIBIORESIST programme could be to concentrate efforts on those GPs identified as most amenable to modifying their prescribing practices. Therefore, to increase trust in A&F, we included not only the health insurance logo on the feedback but also the logos of other trusted partners (e.g. the Grand Est Regional Antibiotic Stewardship Center, *Centre Régional en Antibiothérapie*—CRAtb).

### Mechanisms underpinning the impact of feedback interventions on GPs’ prescribing practices

Given that barriers were predominantly observed during the comprehension and acceptance stages among the subset of GPs whose prescribing patterns deviated most markedly from the established guidelines, their reluctance to engage with A&F highlighting inappropriate practices might be attributed to three hypothetical mechanisms.

The first involves a genuine lack of understanding of the proxy indicators, attributable either to limited statistical literacy and/or excessive complexity of the feedback content.

The second mechanism we hypothesized, grounded in Festinger’s theory of cognitive dissonance,^37^ stems from the fact that despite their potential good understanding of the proxy indicators, GPs seem to experience a conflict between their internal belief in prescribing appropriately and the external A&F, revealing non-conformance with guidelines. According to Festinger, cognitive dissonance creates psychological discomfort that individuals are motivated to resolve, typically through one of two pathways: either by changing their attitudes or by modifying their behaviour to achieve consistency. In this context, GPs may resolve this dissonance by rejecting the A&F content (e.g. by opposing the A&F proxy indicators that did not adequately account for their specific context of practice) or the guidelines themselves rather than adjusting their prescription practices as intended.

The third mechanism we hypothesized occurs when GPs acknowledge the A&F and seem to agree with the scores but remain apprehensive that strict adherence to guidelines may jeopardize patient safety, especially in atypical or complex clinical scenarios in which withholding antibiotics is perceived as risky. Similar mechanisms have been reported elsewhere with a quantitative^38^ and a qualitative approach.^39^

These findings correspond closely to the feedback cycle delineated in the CP-FIT framework, wherein the stages of receipt, comprehension and acceptance of the feedback are pivotal for eliciting the desired prescribing practice changes.^19^ Furthermore, the US CDC emphasizes that optimizing antibiotic use requires ABS programmes that combine A&F with complementary strategies from different domains, such as education, monitoring, responsibility, ABS actions, drug expertise, leadership commitment and accountability.^16^ We hypothesize that the co-interventions planned within the ANTIBIORESIST programme—including an online repository of training modules, an ABS toolbox and academic detailing—constitute critical levers to enhance both the acceptability of the feedback and its ability to foster practice changes in the feedback intervention within the CP-FIT cycle.

Some limitations must be acknowledged. A number of 10 participants may appear low. However, for narrow objectives, such as assessing the item wording, the acceptability of formatting or the ease of implementation, a sample of 10 participants or fewer may adequate.^25–27^ Conducting interviews with volunteers may also have introduced selection bias. The findings may therefore not reflect views of all GPs least compliant with national antibiotic prescribing guidelines who received the A&F intervention, but perhaps those of GPs who were more interested in ABS.

Additionally, the participants were not re-interviewed to validate the findings. However, a synthesis of the results was subsequently provided to a stakeholder group constituted by two GPs and one infectious disease specialist affiliated with the Grand Est regional ABS centre and three GPs from local multidisciplinary ABS teams. The group held two meetings, after which they concluded that the analysis was complete and did not require further analysis.

### Conclusions

We identified 22 elements that may influence the effectiveness of A&F in improving antibiotic prescribing practices among GPs, demonstrating the importance of aligning performance indicators with guidelines as well as the cognitive accessibility and credibility of the A&F provider and the delivery modalities. These findings provide actionable insights for refining (i) A&F features, (ii) the characteristics of the targeted GPs and (iii) the implementation process. Additionally, they offer practical guidance for designing A&F interventions that are likely to strengthen their impact on antibiotic prescribing in primary care when coupled with supportive co-interventions.

## Supplementary Material

dlag197_Supplementary_Data

## Data Availability

The datasets used and/or analysed during the current study are available from the corresponding author upon reasonable request.

## Supplementary data

Additional Files 1, 2, 3 and 4 are available as Supplementary data at *JAC-AMR* Online.
