# Supplementary material for: General practitioners’ perspectives on barriers and facilitators of the implementation of the ANTIBIORESIST antibiotic stewardship audit and feedback intervention: a qualitative interview study among high prescribers
Source: JAC Antimicrob Resist. 2026 Sep 8;8(5):dlag197. doi: 10.1093/jacamr/dlag197 (PMC13551614; doi:10.1093/jacamr/dlag197)
Supplement: Supplementary file 1 [file dlag197_supplementary_data.zip › Additional_file_2_PI.docx]

**Additional file 2.** Description of the eleven proxy indicators

| **Proxy indicator** | **Definition** | **Target population** | **Target value** |
| --- | --- | --- | --- |
| **Estimated duration of antibiotic prescriptions >7 days** | (Number of prescriptions for amoxicillin, amoxicillin-clavulanate, cefuroxime, cefpodoxime, roxithromycin, clarithromycin, pristinamycin, and nitrofurantoin lasting more than 7 days) / total number of prescriptions for these 8 antibiotics | all patients | < 10% |
| **Seasonal variation of total antibiotic prescriptions** | ((Number of prescriptions of antibiotics during the cold-weather season (January–March and October–December) / number of consultations during the cold-weather season]) / (Number of prescriptions of antibiotics during the hot-weather season (April–September) / number of consultations during the same period) − 1} × 100 | all patients | < 20% |
| **Seasonal variation of quinolone prescriptions** | (((Number of fluoroquinolone prescriptions in winter (January–March and October–December) / number of consultations during the same period) / (number of fluoroquinolone prescriptions in non-winter (April–September) / number of consultations during the same period)) – 1) × 100 | ≥ 16 years old | < 10% |
| **Antibiotic prescriptions against urinary tract infections in women** | (Number of prescriptions of nitrofurantoin + pivmecillinam + fosfomycin-trometamol) / number of fluoroquinolone prescriptions | females ≥ 16 years old | > 5 |
| **Amoxicillin/amoxicillin-clavulanate prescriptions** | Number of amoxicillin prescriptions / number of amoxicillin-clavulanate prescriptions | ≤ 65 years old | > 3 |
| **Amoxicillin/second-line antibiotics prescriptions** | Number of amoxicillin prescriptions / (number of amoxicillin-clavulanate + fluoroquinolone + cephalosporin + macrolide and related prescriptions) | ≤ 65 years old | > 3 |
| **Repeated prescription of quinolones** | (Number of fluoroquinolone prescriptions among patients who received a fluoroquinolone in the previous six months) / total number of fluoroquinolone prescriptions | males and females ≥ 16 years old | < 10% |
| **Prescriptions of pristinamycin and macrolides** | (Number of pristinamycin + macrolide prescriptions) / total number of antibiotic prescriptions | all patients | < 10% |
| **Prescriptions of not indicated antibiotics** | (Number of prescriptions of moxifloxacin, spiramycin-metronidazole, and cefaclor) / total number of antibiotic prescriptions | all patients | < 0.5% |
| **Co-prescription of antibiotic and systemic non-steroidal anti-inflammatory drugs** | Number of antibiotics and non-steroidal anti-inflammatory drugs co-prescribed on the same day / total number of antibiotic prescriptions | all patients | < 5% |
| **Co-prescription of antibiotic and systemic corticosteroids** | Number of antibiotics and systemic corticosteroids co-prescribed on the same day / total number of antibiotic prescriptions | all patients | < 5% |
