# Supplementary material for: General practitioners’ perspectives on barriers and facilitators of the implementation of the ANTIBIORESIST antibiotic stewardship audit and feedback intervention: a qualitative interview study among high prescribers
Source: JAC Antimicrob Resist. 2026 Sep 8;8(5):dlag197. doi: 10.1093/jacamr/dlag197 (PMC13551614; doi:10.1093/jacamr/dlag197)
Supplement: Supplementary file 1 [file dlag197_supplementary_data.zip › Additional file 4.docx]

**Additional file 4.** Detailed characteristics of the included GPs.

| Pseudonym | Sex (Male/Female) | Age (years) | Professional experience (years) | Location (Rural/Semi-Urban/Urban) |
| --- | --- | --- | --- | --- |
| GP1 | Male | 54 | 23 | Semi-urban |
| GP2 | Male | 56 | 26 | Rural |
| GP3 | Male | 60 | 34 | Semi-urban |
| GP4 | Female | 33 | 6 | Semi-urban |
| GP5 | Male | 46 | 18 | Rural |
| GP6 | Male | 65 | 37 | Rural |
| GP7 | Female | 46 | 18 | Rural |
| GP8 | Male | 39 | 12 | Urban |
| GP9 | Male | 66 | 36 | Urban |
| GP10 | Male | 57 | 25 | Rural |

Notes: GP: general practitioner; N/A: not available
